# Autonomous artificial intelligence in pediatric radiology: the use and perception of BoneXpert for bone age assessment

**DOI:** 10.1007/s00247-022-05295-w

**Published:** 2022-02-28

**Authors:** Hans Henrik Thodberg, Benjamin Thodberg, Joanna Ahlkvist, Amaka C. Offiah

**Affiliations:** 1 Visiana, Hørsholm, Denmark; 2 Nyköping Hospital, Nyköping, Sweden; 3 Department of Radiology, Academic Unit of Child Health, University of Sheffield, Damer Street Building, Western Bank, Sheffield, S10 2TH UK

**Keywords:** Artificial intelligence, Bone age, Children, Hand, Musculoskeletal, Radiography, Wrist

## Abstract

**Background:**

The autonomous artificial intelligence (AI) system for bone age rating (BoneXpert) was designed to be used in clinical radiology practice as an AI-replace tool, replacing the radiologist completely.

**Objective:**

The aim of this study was to investigate how the tool is used in clinical practice. Are radiologists more inclined to use BoneXpert to *assist* rather than *replace* themselves, and how much time is saved?

**Materials and methods:**

We sent a survey consisting of eight multiple-choice questions to 282 radiologists in departments in Europe already using the software.

**Results:**

The 97 (34%) respondents came from 18 countries. Their answers revealed that before installing the automated method, 83 (86%) of the respondents took more than 2 min per bone age rating; this fell to 20 (21%) respondents after installation. Only 17/97 (18%) respondents used BoneXpert to completely replace the radiologist; the rest used it to assist radiologists to varying degrees. For instance, 39/97 (40%) never overruled the automated reading, while 9/97 (9%) overruled more than 5% of the automated ratings. The majority 58/97 (60%) of respondents checked the radiographs themselves to exclude features of underlying disease.

**Conclusion:**

BoneXpert significantly reduces reporting times for bone age determination. However, radiographic analysis involves more than just determining bone age. It also involves identification of abnormalities, and for this reason, radiologists cannot be completely replaced. AI systems originally developed to *replace* the radiologist might be more suitable as AI *assist* tools, particularly if they have not been validated to work autonomously, including the ability to omit ratings when the image is outside the range of validity.

**Supplementary Information:**

The online version contains supplementary material available at 10.1007/s00247-022-05295-w.

## Introduction

In this article, we define artificial intelligence (AI) as software that automates a cognitive task. Since 2012, there has been dramatic progress in AI technology, in particular in image analysis [1]. This has caught the attention of the news media, which often overestimates and sometimes demonizes AI, leading to heated debate about ethics as well as unmet promises [2].

There are intense discussions about how AI might affect the future of radiology, raising questions as to whether young doctors will be less inclined to train as radiologists and whether AI is dangerous, for example [3]. There is consensus in the radiology community that AI will not replace radiologists but that radiologists who use AI will replace those who do not [4]. However, many also believe that at least some radiology tasks will be completely taken over by AI, possibly operated by the treating physician [5].

Following van Ginneken [5], we subdivide AI systems into three types:

- AI-assist: AI that assists the radiologist,
- AI-replace: AI that replaces the radiologist and
- AI-extend: AI that derives image information that goes beyond what a human would extract routinely.

**Fig. 1:**
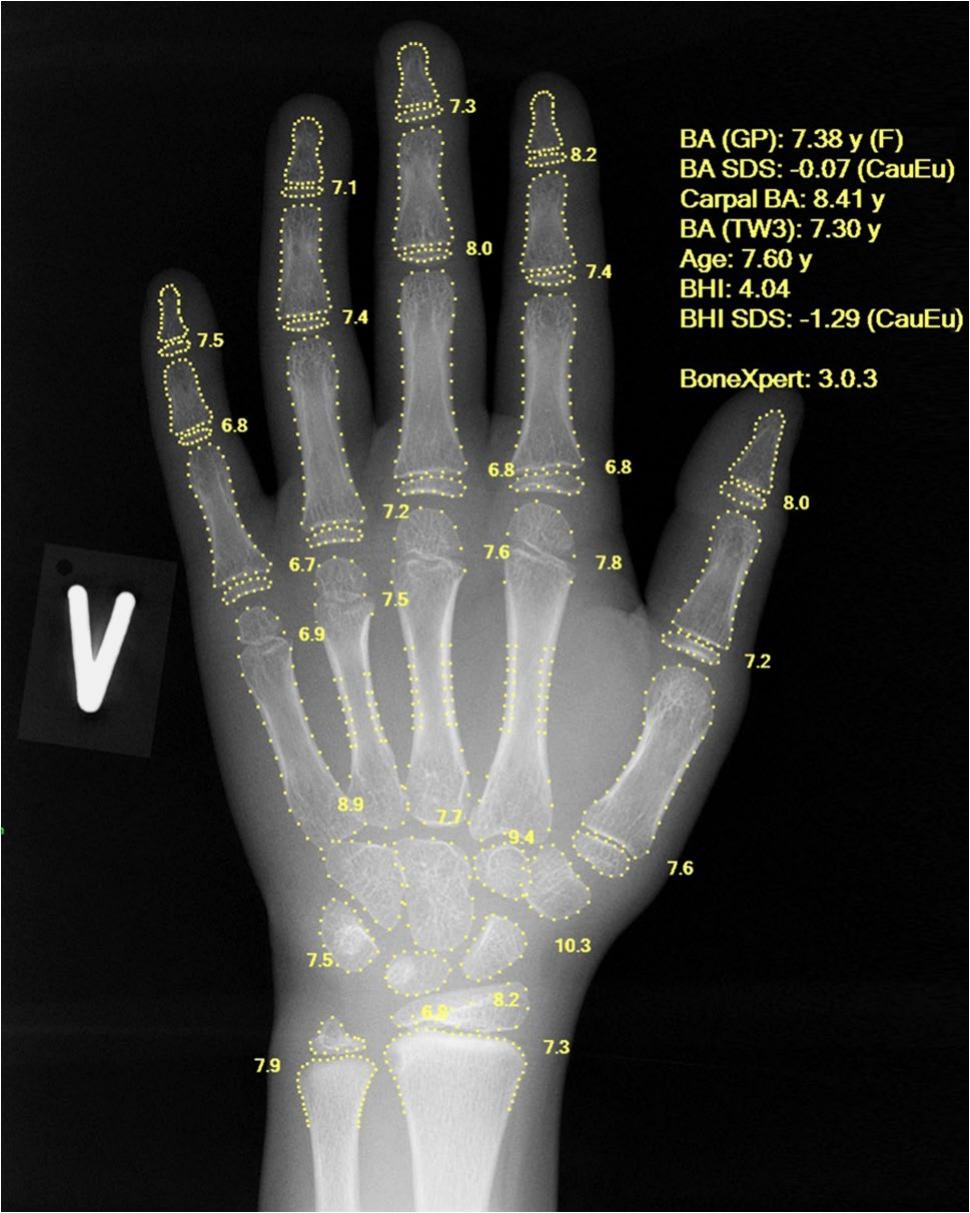
Dorsopalmar left hand radiograph in a 7.6-year-old girl following bone age assessment by BoneXpert. The output of the artificial intelligence (AI) system is an annotated Digital Imaging and Communications in Medicine (DICOM) file placed in the same study in the hospital’s picture archiving and communications system (PACS) as the original image. The algorithm has located the borders of the bones and assigned a Greulich and Pyle (GP) bone age to each of them. The average bone age (BA) for the 21 tubular bones is reported as “BA (GP): 7.38 y (F),” where the F indicates female gender, as taken from the DICOM header. The next line reports a bone age standard deviation score (SDS) of –0.07, which means that the bone age is 0.07 standard deviations below what is expected at that chronological age. Chronological age is indicated below the bone age SDS as 7.60 years (computed from the birth and study dates in the DICOM header). The remaining reported numbers are: carpal BA = the average bone age in the seven carpals, BA (TW3) = Tanner and Whitehouse version 3 bone age, BHI = bone health index, and its SDS relative to girls with the same bone age

In this paper we investigated the adoption of AI in radiology, using the example of bone age assessment. Bone age is a measure of the maturity of the bones, and it is usually assessed from a hand and wrist radiograph (Fig. 1). The bone age, expressed in years, is the age at which half of the children in a reference population have attained the observed degree of maturation based on features such as the relative width of the epiphyses.

The most common bone age method is Greulich and Pyle [6], which is based on a reference population of middle class children in the USA between 1931 and 1942.

The BoneXpert method (Visiana, Hørsholm, Denmark) for automated determination of bone age was launched in 2009 by the company Visiana [7]. The intended use is to completely replace the human rating of bone age, and in accordance with this, all clinical investigations during its development were performed with BoneXpert working as a standalone reader. The image analysis is based on traditional machine-learning methodology and involves prediction of bone age based on shape, intensity and texture scores derived from principal component analysis. The method attempts to locate almost all the bones in the hand and wrist (no sesamoid bones are included), as shown in Fig. 1, and determines an independent bone age value for each. A bone is rejected if its visual appearance falls outside the range covered in the machine learning process, or if its bone age value deviates by more than a predefined threshold from the average bone age determined from all the tubular bones. The threshold is set at 2.4 years for patients older than 7 years, then decreases linearly to 1.2 years at birth. The final bone age result is computed as an average of the accepted bones. The method rejects the image if there are fewer than eight accepted bones, to avoid the risk of the automated rating being wrong. This internal validation process is considered crucial for an AI-replace system. The software produces an annotated Digital Imaging and Communications in Medicine (DICOM) file (Fig. 1).

Although BoneXpert is classified as AI-replace, it can be used as an AI-assist tool, depending on the preference of the user. BoneXpert also plays an AI-extend role, in that it calculates the bone health index (a measure of the cortical thickness of the second to fourth metacarpal shafts) and compares the patient’s bone health index to that of a healthy population of the same bone age and gender.

Several authors have assessed the diagnostic accuracy of BoneXpert in clinical practice [8, 9] and in retrospective studies involving a number of disorders, e.g., short stature [10, 11], precocious puberty [12] and congenital adrenal hyperplasia [13], and in different ethnic populations [8, 14–16]. These studies have found an average accuracy (root mean square error) of 0.72 years [17]. In 2019, the software was updated (BoneXpert version 3), taking advantage of increased availability of training data, increasing the accuracy to a root mean square error of 0.63 years relative to a single rater and 0.45 years relative to the average of six raters, thus clearly surpassing the accuracy of humans [18, 19]. BoneXpert was the first AI-replace radiology system to be marketed, and as of April 2021 it was in use by 200 radiology departments, mainly in Europe, together performing more than 100,000 analyses per year.

There are several reasons why bone age assessment is well-suited to complete automation:

- It is not a high-risk task. Making an error in bone age assessment in a clinical setting [20, 21] might have less serious consequences than, for example, missing a cancer (although of course not always).
- Bone age can be determined from each of 21 bones of the hand and wrist (excluding the carpal bones). This redundancy allows the exclusion of outlier bones, thereby making an automated assessment very robust to errors in single bones.
- The anatomy of the 21 tubular bones (phalanges, metacarpals and distal radius and ulna) appears very clearly on the image, with no overlapping bones and very little positional variation. This makes it easy to develop an algorithm that can segment the bones reliably. In the subsequent image interpretation, the bones are always seen in the same projection, i.e. positional variation is only a small confounding factor.
- Determination of bone age by radiologists is subjective, using visual comparison with the Greulich and Pyle reference atlas. Often there is no perfect match in the atlas; instead, one must look for the most similar reference image. This is a complex cognitive task requiring expertise. Computers, on the other hand, have an advantage in that they can convert the data from the images to numbers and thus assess bone age as a continuous variable.
- There are many bones, and it is by rating them all carefully that one obtains the highest accuracy, which takes a lot of time, if done manually.

Because of the last two points (d and e), some radiologists are less enthusiastic about the task of bone age determination, and it might be delegated to junior radiologists. The lack of popularity of bone age reporting can lead to delays in reporting.

The groundwork for automated bone age rating has been accumulating for decades. Tanner, a significant contributor to the field of bone age assessment [22, 23], presented a working prototype of a semi-automated bone age system as far back as 1989, and he made this statement about computerized bone age assessment [24]: “Surely this is the way forward, eliminating the all-too-fallible rater entirely.”

Twenty years passed before Tanner’s vision of AI-replace for bone age determination was realized with the introduction of BoneXpert in 2009, presenting a unique opportunity for clinical radiologists to experience the use of an AI-replace system on a day-to-day basis.

The aim of this study was to investigate how the automated system is used in clinical practice: to what extent it replaces the radiologist, whether it allows time saving, and what features might enhance radiologists’ trust in the system.

## Materials and methods

Data collection was conducted using an online questionnaire implemented using SurveyMonkey (SurveyMonkey, San Mateo, CA). The survey was first sent out by email on 15 June 2020 and again on 25 October 2021. Recipients were chosen based on the portfolio of BoneXpert customers, and the inclusion criteria were:

1. The recipient should be an active user of BoneXpert.
2. Job title should be “radiologist” or “head of radiology.”
3. The country should be in the European Union, or the United Kingdom, Norway, Iceland or Switzerland (regions for which BoneXpert has received the CE Mark).

This resulted in 282 email addresses distributed over 149 hospital departments and clinics. Three additional reminder emails were sent out after the initial email if no answer had been received.

The survey consisted of eight multiple-choice questions (Online Supplementary Material 1). The first four questions were designed to investigate the practical use of the software: the time saved by its use, the frequency with which the BoneXpert reads were overruled by radiologists and whether it was used as AI-replace or AI-assist. Although not directly related to bone age assessment, for the sake of completion, we included a fifth question on the functionality of the software as related to its ability to determine the bone health index. The final three questions assessed the radiologists’ subjective perceptions of the software: what feature they valued most about its functionality and trustworthiness and whether they would recommend BoneXpert to others. The survey also included the option for open answers (“other — please specify below”) to capture answers outside the set options. The license conditions for BoneXpert explicitly state that use for assessment of age for asylum seekers is not permitted, and neither is the use of bone age to determine undocumented chronological age endorsed by the European Society of Paediatric Radiologists [25]. In accordance with this, such use was excluded from this survey.

Written informed consent and institutional review board approval were not required for this study because it involved no patients. Participation in the questionnaire was voluntary. Respondents were informed that the purpose of the questionnaire was to produce a publication, and that their anonymity was guaranteed.

Statistical analyses were performed using MATLAB (MathWorks, Natick, MA). The *P*-values were computed using bootstrapping (resampling the 97 respondents a million times with replacement, see chapter 10 of [26]). *P*<0.05 was significant. Only the data from respondents who answered all eight questions were analyzed.

## Results

### Questionnaire responses

**Fig. 2:**
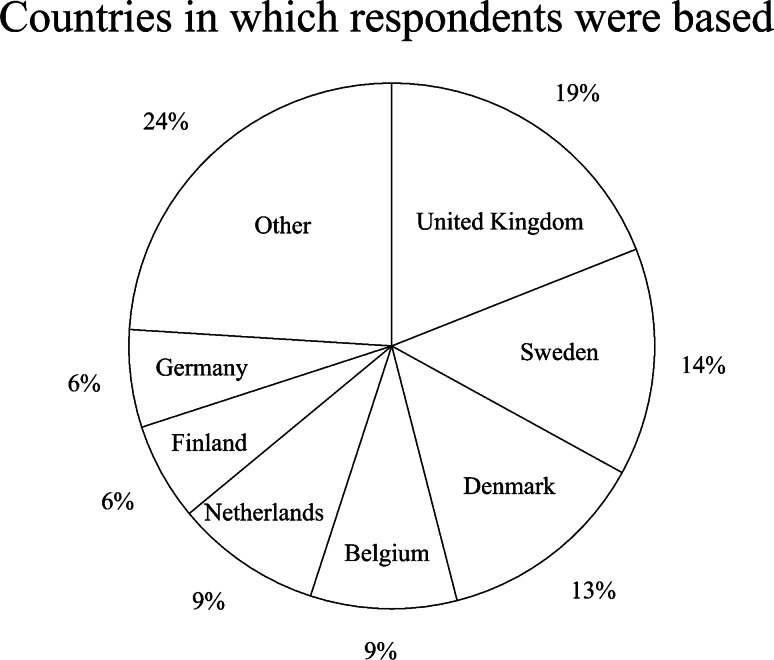
Chart depicts countries in which respondents were based. The group “other” includes four respondents in Italy; three each in Norway and Switzerland; two each in Iceland, Slovenia, Czech Republic and Austria; and one each in Estonia, Luxembourg, Greece and France

Of the 282 recipients and 149 departments, 97 (34%) recipients responded, representing 80 (54%) departments and 18 countries (Fig. 2). Thus, the average number of respondents per department was 1.21. Three departments submitted three responses: Alder Hey (UK), Odense (Denmark) and Linköping (Sweden); the rest submitted one or two each. The annual number of BoneXpert analyses varied from 20 to 3,500; the median annual number across the 97 responses was 300. The age distribution for bone age assessment across the responding departments was not captured by the survey.

### Questions and answers

Tables 1, 2, 3 and 4 present six of the eight questions and the responses to them. The remaining two questions (5 and 8) were relatively straightforward and therefore not tabulated.

**Table 1:** Time spent on bone age assessments

Q1 - Before you got BoneXpert, how long did you spend on each bone age evaluation?	
No time – the rating was done by someone else	5%
Less than 2 min	9%
Between 2 and 5 min	49%
More than 5 min	37%
Q2 - Since you got BoneXpert, how much time do you use to do a bone age evaluation?	
No time - I no longer look at the image	17%
Less than 2 min	62%
Between 2 and 5 min	20%
More than 5 min	1%

**Table 2:** AI-Replace versus AI-Assist (Question 3)

Q3 - Would you let BoneXpert take over bone age rating completely? (select one or more items)	
Yes – this is how we use it today	33%
No - I need to look at the image for signs of abnormalities, e.g., skeletal dysplasias or Turner syndrome	60%
No - I want to ensure that the bone age is done correctly	14%
I can’t - For legal reasons, every image must be seen by a radiologist	13%
I can’t - For economical/reimbursement reasons, every image must be seen by a radiologist	2%
[Other]	3%
Q4 - How often do you override the bone age value provided by BoneXpert?	
Never	40%
Less than 5% of the cases	43%
5–25% of the cases	5%
More than 25% of the cases	4%
I do not know / I cannot answer this question	8%

**Table 3:** How valuable do you find the different features of BoneXpert? (question 6)

	Highly valuable	Valuable	Neither valuable nor worthless	Worthless	Completely worthless
BoneXpert eliminates the human rater variability and gives a standardized bone age value	59%	38%	3%	0%	0%
BoneXpert saves time for the radiologist	64%	25%	10%	0%	1%
BoneXpert takes away a tedious and strenuous task	42%	41%	12%	3%	1%
BoneXpert has a very user-friendly integration with the PACS workflow	46%	36%	14%	2%	1%
With BoneXpert, the referring physician receives the results sooner	37%	33%	20%	4%	6%
The ability to generate a PDF report	15%	24%	42%	9%	8%

*PACS* picture archiving and communications system, *PDF* portable document format

**Table 4:** Aspects of BoneXpert earning respondents’ trust in the tool (question 7)

Given situations	BoneXpert has not taken over bone age rating completely *n*=65 (67%)	BoneXpert has taken over bone age rating completely *n*=32 (33%)	*P-*value^a^
Regulatory conformance, such as CE Mark, and an ISO 13485-based quality assurance system (and later: FDA clearance)	43%	56%	0.12
The good performance data and the 20 peer-reviewed publications documenting these data	69%	75%	0.27
Support from the vendor (Visiana)	20%	19%	0.43
The system is used in many other hospitals	35%	50%	0.09
The system explains how it arrives at the assessment by showing the outline and bone age of each used bone	34%	25%	0.18
The system automatically rejects an image if it is not certain about its interpretation	48%	44%	0.36
My department has performed its own validation of the system	20%	6%	**0.02**

The table summarizes responses to the question, “Which of the following aspects are most important for trusting BoneXpert’s bone age determination?” Here, the respondents were segmented according to whether the automated method served an AI-assist or an AI-replace function within individual departments

*AI* artificial intelligence, *FDA* United States Food and Drug Administration

^a^*P*-value <0.05 is significant (bold)

### Time savings (Q1 and Q2 — Table 1)

**Fig. 3:**
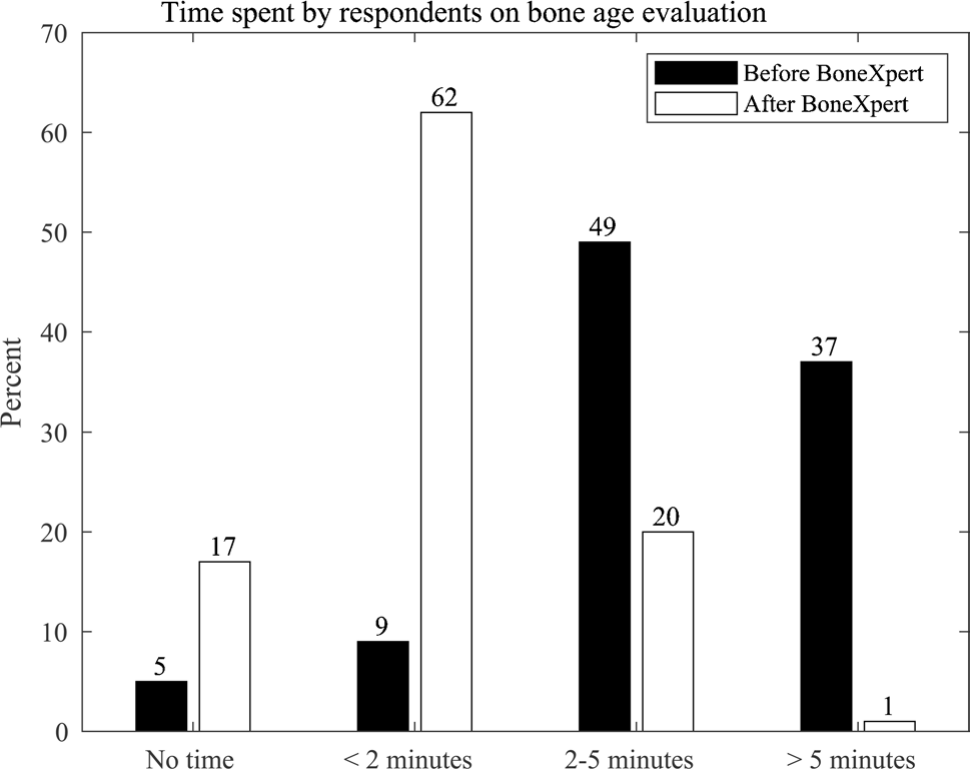
Graph shows time spent by respondents on bone age evaluation. The percentage of respondents taking less than 2 min to report each bone age radiograph rose from 14% before BoneXpert was installed to 79% after, with a drop in those taking more than 5 min from 37% to 1% pre and post BoneXpert installation, respectively. The figure shows that the number of radiologists not assessing bone age at all (“no time”) rose from 5% before BoneXpert to 17% after

There was a manifest change in bone assessment workflow after the installation of BoneXpert (Fig. 3); 83 (86%) reported that bone age assessments took longer than 2 min per patient before BoneXpert, compared to 20 (21%) after installation.

### Artificial intelligence (AI)-replace or AI-assist? (Q3 — Table 2)

Responses to Q3 revealed that in practice, usage of BoneXpert covers the full spectrum from assisting to replacing the reporter. At one end of the spectrum, 32 (33%) responders allowed the automated method to calculate bone age completely by itself. At the other end of the spectrum, 14 (14%) reviewed every report to ensure that bone age was determined correctly. A majority 58 (60%) did not allow BoneXpert to take over bone age ratings completely because of the need to review the images for signs of disease, and 13 respondents (13%) believed that reviewing the image was a legal obligation.

**Fig. 4:**
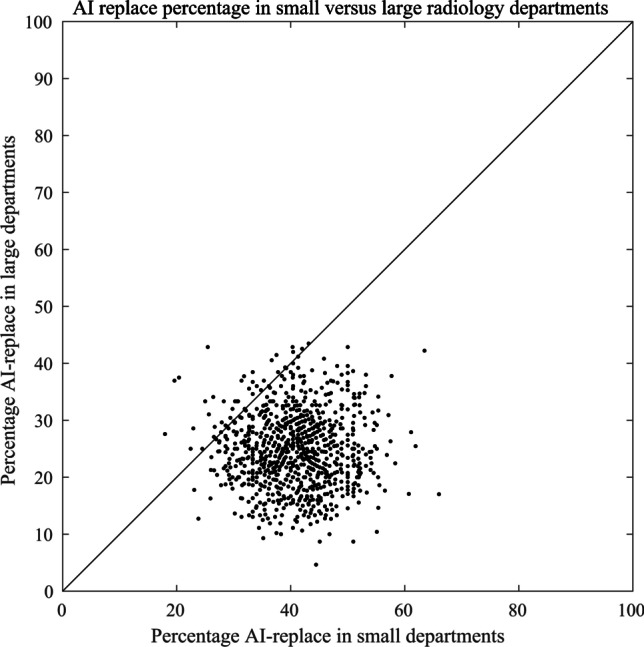
Artificial intelligence (AI)-replace percentages in small versus large departments. Resampling (bootstrapping) of the observed survey data shows that the respondents from smaller departments were significantly more inclined to use the method as AI-replace than the respondents from larger departments (*P*-value=0.048)

We further explored the AI-replace/AI-assist question by dividing the 97 responses into the 49 from smaller sites that were doing less than or equal to the median number of analyses per year (300) and 48 from larger sites that were doing more than the median number. In the small-site group, AI-replace (answering yes to Q3) reached 41%, while in the large-site group it was only 25% (Fig. 4). This difference just reached statistical significance (*P*=0.048).

### Over-ruling the software (Q4 — Fig. 5)

Considering the responses to Q4, 39 (40%) respondents had never overruled the BoneXpert read, while 9 (9%) had overruled it in more than 5% of cases.

**Fig. 5:**
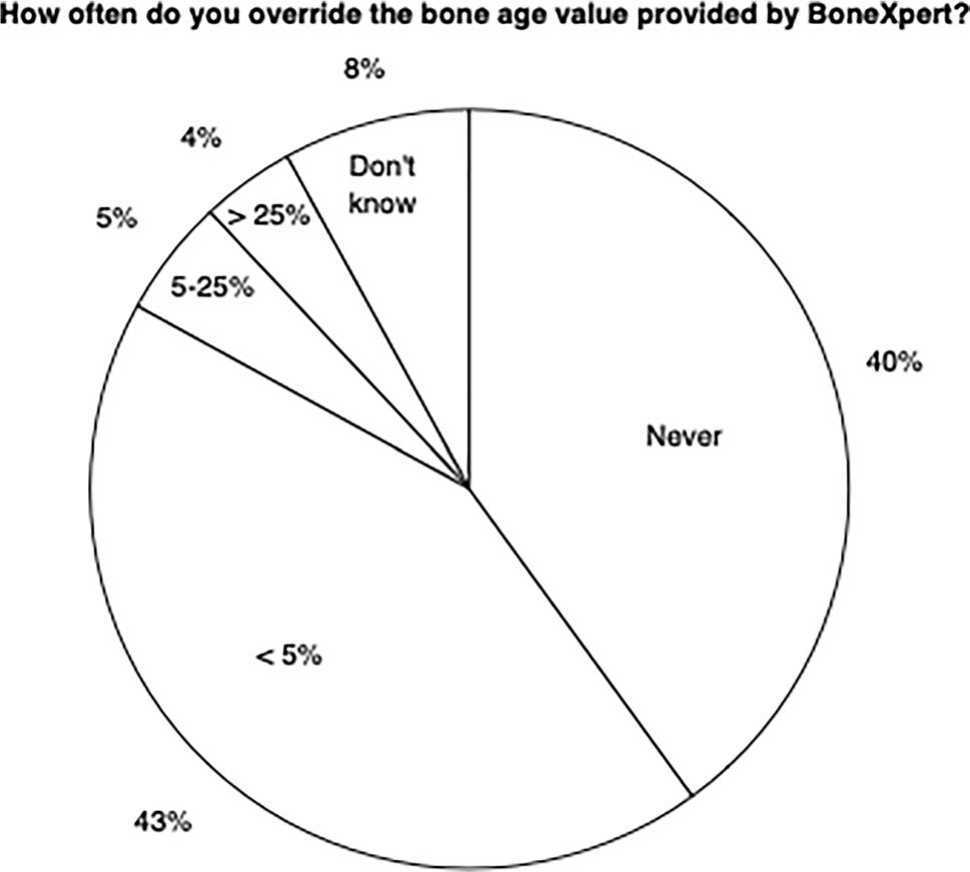
Distribution of radiologists according to how often they overrode the automated rating. The majority of respondents (83%) overrode BoneXpert in less than 5% of bone age radiographs

### Bone health index (Q5)

A third (33) of responding radiologists found the bone health index (BHI) to be clinically useful, 16 (17%) did not find it useful, while the remaining 48 (49%) were unsure.

### Value (Q6 — Table 3)

The numbers of respondents finding the time savings and elimination of observer variability either valuable or highly valuable were 86 (89%) and 94 (97%), respectively. A significant number, 68 to 81 (70% to 83%), also found that BoneXpert’s utility for taking away a tedious task, integrating with PACS and getting results earlier to be valuable or highly valuable features.

### Trust (Q7)

Clinical evaluation of data (i.e. performance data and peer-reviewed publications) was the most important factor in generating trust in AI, having been selected by 69 (71%) respondents. Regulatory clearance (i.e. the CE Mark) and automatic rejection of inadequate images were the other important aspects, selected by 47 (48%) and 45 (46%) respondents, respectively.

To further evaluate the answers to Q7, we split the respondents into two groups: the group AI-replace, defined as those answering yes to Q3 (“Would you let BoneXpert take over bone age rating completely?”), and the group AI-assist, defined as the remaining respondents. Table 4 compares the answers to Q7 between the 32 (33%) group AI-replace respondents and the 65 (67%) others and shows that the only statistically significant difference between them was that those radiologists from departments that had performed their own self-validation of BoneXpert were more likely to also review the radiographs.

### Recommendations (Q8) — would you recommend BoneXpert to another radiologist?

With regard to question 8, 99% of the respondents answered yes and 1% answered no.

## Discussion

There are two opposing views on the future role of AI in radiology, exemplified by Langlotz [4], who argued for AI-assist, and van Ginneken, [5], who argued for AI-replace for at least some exams.

In this paper, we report how a widely used AI-replace system has altered the clinical workflow within radiology departments across Europe. Although responses were given by individual radiologists who use BoneXpert, we are assuming these data represent the usage pattern of radiologists in general. These data are therefore an attempt to summarize usage among radiologists rather than across institutions. Given that there were only 97 respondents, what follows is a qualitative discussion of the use of AI in radiology.

BoneXpert was designed as an AI-replace system based on the conjecture that bone age rating is particularly well suited for such a system. However, the survey showed that 82% of responding radiologists were still performing some degree of assessment of the radiographs, even though they had the automated method installed. This suggests an AI-assist role for BoneXpert. The survey revealed that the situation is even more complex, and “reading of bone age hand and wrist radiographs” really consists of two tasks:

1. a quantitative task of bone age rating, per se, and
2. a general, qualitative diagnostic task.

The first task is mechanistic, time-consuming and based on several maturity indicators such as “width of epiphysis” and “degree of epiphyseal fusion” of each bone. For the performance of this task, it has even been considered an advantage not to know the context, such as the diagnosis, results of previous ratings, or chronological age of the patient [27].

The second task, that of excluding underlying pathology, is qualitative, but comparatively quick for radiologists to perform and is the sort of analysis in which they excel; it requires a breadth of experience, understanding of the context (e.g., patient history), and the ability to generalise from skeletal radiology at other sites [28]. While AI systems might be trained to perform these tasks, a sufficiently large data set to allow specific diagnosis of skeletal dysplasias (for example) would be difficult to acquire.

We found that in the first task, AI has largely replaced radiologists, but to a varying degree. This is illustrated in Fig. 3, which shows that with the introduction of the automated method, reporting times were reduced from typically more than 2 min to typically less than 2 min, and in Fig. 5, which shows that automated reading is only rarely overridden by the radiologist, despite the majority reviewing the radiographs. We can summarize our findings by saying that after the introduction of the automated method, radiologists are still reading the radiographs, but mainly to exclude radiologic findings that might indicate an underlying disorder.

Table 3 presents the features of the automated method most valued by the radiologist, with the highest ranked feature being that “BoneXpert eliminates the human rater variability and gives a standardized bone age value.” This aspect has value for the patients, because the better precision of the automated method has clinical significance when following patients longitudinally. In these situations, the clinicians want to assess the bone age *increments*, and these increments are determined less precisely with human ratings because of rater variability, something that severely limits the usefulness of manual bone age ratings, e.g., during growth hormone therapy [29, 30].

Table 4 presents the aspects that respondents reported earn their trust in the automated method. Publications and performance data ranked higher than regulatory conformance, which is another way of saying that radiologists have greater faith in transparent peer-reviewed scientific publications than in the process of CE-marking. In Table 4, we singled out the AI-replace group, i.e. the 32 (33%) respondents who leave bone age rating completely to the automated method. They tended toward being more trusting of regulatory conformance than the complementary AI-assist group, although this finding was not statistically significant (*P*=0.11). A greater number, 13 (20%) of the AI-assist group, had performed a validation of the automated method in their own department, compared to the AI-replace group at only 2 (6%). We might characterize the AI-assist group as being more aware of the limitations of BoneXpert to identify pathology. Interestingly, our results also suggest that smaller departments are more inclined to use the method as AI-replace. Perhaps this reflects patient populations, such that children with skeletal dysplasias/bone pathology are more likely to be seen in larger specialist hospitals.

We have seen that the automated method is most often used with some supervision from a human reader. Despite this, we feel that there is benefit in the fact that the method has been validated to work autonomously, and that it automatically rejects radiographs not deemed to be suitable for automated rating. The latter functionality serves as a safety measure, drawing the radiologist’s attention to a pathological or quality issue that means the image cannot be read by BoneXpert. However, in a proportion of cases, the machine effectively evaluates the radiographs on its own. This is directly evident in Fig. 5, which shows that 39 (40%) radiologists admitted to *never* overruling BoneXpert, despite being given an alternative answer option, “I override BoneXpert in less than 5% of cases.” This finding emphasizes the importance of validating AI systems to be able to work independently. AI systems should include safety measures that analyze the adequacy of the input data (image quality, anatomy, etc.) and only generate conclusions when appropriate for the AI system, i.e. within its range of validity. Poorly underpinned analyses might otherwise go unnoticed by the less observant human. We found some evidence for this point in Q7, wherein 45 (48%) radiologists responded that the fact that “The system automatically rejects an image if it is not certain about its interpretation” was important for trusting the system. This property is perhaps underestimated by both users and industry. Besides BoneXpert, the authors are aware of two other automated bone age systems, VUNO Med-Bone Age (VUNO, Seoul, Korea) and IB Lab PANDA (ImageBiopsy Lab, Vienna, Austria) [31]. Neither includes such a sophisticated mechanism for image validation. It would be interesting to see how these are used in clinical practice compared to BoneXpert.

It is our opinion that an AI-assist system should not be approved for clinical use based solely on studies where it is used as AI-assist. There must also be extensive studies demonstrating that its autonomous performance is at least as good as that of a radiologist, and these studies should include inadequate/poor-quality images, which the system should either rate correctly or not rate at all [18]. We believe that this aspect of AI-replace/AI-assist has not been sufficiently studied in the literature.

This study has limitations. First, the response rate was only 34%; higher response rates would have given more representative/reliable data. Second, in our own clinical practice, we find it acceptable that BoneXpert rejects approximately 3% of radiographs (because of abnormal bone appearances or poor positioning), but it would have been useful to question users about what rejection rates they experienced and whether they found this to be acceptable. Third, the survey only included hospitals that had already purchased a license, and so were more likely to have a positive view of the software (we could have investigated this potential bias by asking respondents whether they were directly involved in the decision to purchase BoneXpert). The risk of bias would presumably have been reduced had we installed the program for free in participating hospitals and questioned radiologists after installation, i.e. if we had performed a prospective rather than an observational study. Fourth, we assumed that each radiologist would perform bone age assessment across the entire pediatric age range, but this might not be the case and could impact the mode by which BoneXpert is used (AI-replace or AI-assist). Finally, we conducted the survey and present results at an individual rather than institutional level. While there might be some repetition of information, we took this approach because we felt that individuals within a department might differ in their use and opinion of new technology.

## Conclusion

The vast majority (82%) of respondents using BoneXpert AI software have not entirely excluded radiologists from the task of bone age determination. This survey illustrates that bone age rating is more complex than just delivering the bone age number. The practice at most institutions is to also assess the images for possible signs of underlying disease. We would encourage this practice and as such discourage endocrinologists (and doctors from other specialties) from bypassing the radiologist entirely because there could be relevant findings beyond bone age and important diagnoses might otherwise be missed.

The introduction of BoneXpert represents an efficient division of labor between machines and humans — each does what they are best at, and they do it quickly and safely. This is an example of good use of AI in radiology: workflow changes for the better, the accuracy and precision of the assessment increases [32], and the radiologist’s time is freed to perform more complex imaging tasks. There is reassurance in the fact that the method has been validated to be able to work autonomously, including the ability to omit ratings when the image is outside the range of validity.

## Supplementary Information

ESM 1 (PDF 84 kb)
