# Supplementary material for: Autonomous artificial intelligence in pediatric radiology: the use and perception of BoneXpert for bone age assessment
Source: Pediatr Radiol. 2022 Feb 28;52(7):1338–46. doi: 10.1007/s00247-022-05295-w (PMC9192461; doi:10.1007/s00247-022-05295-w)
Supplement: Supplementary file 1 — (PDF 84 kb) [file 247_2022_5295_MOESM1_ESM.pdf]

BoneXpert

The role of AI in radiology: The case of BoneXpert (EUR)

⊕ PAGE TITLE

Dear BoneXpert user,

The role of AI in radiology is currently being discussed intensely. We would like to contribute to this debate by conducting a survey on the use of BoneXpert. We would be very grateful for your help, as a radiologist, in answering some simple questions.

We will summarize the result as an empirical study of how the BoneXpert method for automated bone age rating is used today in the 150 clinics who have adopted the method in daily clinical use. Respondents will receive a summary report.

Please answer the following questions – your answers are kept confidential.

* 1. Before you got BoneXpert, how long did you spend on each bone age evaluation? 0

- ◯ No time – the rating was done by someone else
- ◯ Less than 2 minutes
- ◯ Between 2 and 5 minutes
- ◯ More than 5 minutes

* 2. After you got BoneXpert, how much time do you use to do a bone age evaluation? 0

- ◯ No time - I no longer look at the image
- ◯ Less than 2 minutes
- ◯ Between 2 and 5 minutes
- ◯ More than 5 minutes

* 3. Would you let BoneXpert take over bone age rating completely? (select one or more items) 0

- ☐ Yes – this is how we use it today
- ☐ No - I need to look at the image for signs of abnormalities, e.g. skeletal dysplasias or Turner syndrome
- ☐ No - I want to ensure that the bone age is done correctly
- ☐ I can't - For legal reasons, every image must be seen by a radiologist
- ☐ I can't - For economical/reimbursement reasons, every image must be seen by a radiologist
- ☐ Other (Please specify)

* 4. How often do you override the bone age value provided by BoneXpert? 0

- Never
- Less than 5% of the cases
- 5-25% of the cases
- More than 25% of the cases
- I do not know / I cannot answer this question

* 5. Do you find the Bone Health Index (BHI), as determined by BoneXpert, to have clinical value? 0

- Yes
- No
- I do not know / I cannot answer this question

* 6. How valuable do you find the different features of BoneXpert? 0

	Highly valuable	Valuable	Neither valuable nor worthless	Worthless	Completely worthless
BoneXpert eliminates the human rater variability and gives a standardized bone age value	○	○	○	○	○
BoneXpert saves time for the radiologist	○	○	○	○	○
BoneXpert takes away a tedious and strenuous task	○	○	○	○	○
BoneXpert has a very user-friendly integration with the PACS workflow	○	○	○	○	○
With BoneXpert, the referring physician receives the results sooner	○	○	○	○	○
The ability to generate a PDF report	○	○	○	○	○

* 7. Currently, there is an active discussion on the ethics of introducing AI in radiology. One concern is whether it is ethical to leave a medical assessment to a computer program.

Which of the following aspects are most important for trusting BoneXpert's bone age determination?

(select at least 1 item) 0

- [ ] Regulatory conformance, such as CE-mark, and an ISO 13485-based quality assurance system (and later: FDA clearance)
- [ ] The good performance data and the 20 peer-reviewed publications documenting these data.
- [ ] Support from the vendor (Visiana) on various questions
- [ ] The system is used in many other hospitals
- [ ] The system explains how it arrives at the assessment by showing the outline and bone age of each used bone
- [ ] The system automatically rejects an image, if it is not certain about its interpretation
- [ ] My clinic has performed its own validation of the system
- [ ] Other (Please specify below)

* 8. Would you recommend BoneXpert to another radiologist? 0

- ( ) Yes
- ( ) No

9. How can we improve BoneXpert?

How well does BoneXpert meet your expectations, and where is there room for improvements?

Please give your answer in the text box below. 0

10. If you have any feedback or comments to the survey, BoneXpert, Visiana or AI in radiology in general, please specify in the text box below.

0

DONE
